# Sweet Taste Antagonist Lactisole Administered in Combination with Sucrose, But Not Glucose, Increases Energy Intake and Decreases Peripheral Serotonin in Male Subjects

**DOI:** 10.3390/nu12103133

**Published:** 2020-10-14

**Authors:** Kerstin Schweiger, Verena Grüneis, Julia Treml, Claudia Galassi, Corinna M. Karl, Jakob P. Ley, Gerhard E. Krammer, Barbara Lieder, Veronika Somoza

**Affiliations:** 1 Department of Physiological Chemistry, Faculty of Chemistry, University of Vienna, Althanstrasse 14, 1090 Vienna, Austria; kerstin.schweiger@univie.ac.at (K.S.); veronika.somoza@univie.ac.at (V.S.); 2 Christian Doppler Laboratory for Taste Research, Faculty of Chemistry, University of Vienna, Althanstrasse 14, 1090 Vienna, Austria; verena.grueneis@univie.ac.at (V.G.); treml.julia@hotmail.com (J.T.); a01447824@unet.univie.ac.at (C.G.); corinna.miriam.karl@univie.ac.at (C.M.K.); 3 Symrise AG, Muehlenfeldstrasse 1, 37603 Holzminden, Germany; jakob.ley@symrise.com (J.P.L.); gerhard.krammer@symrise.com (G.E.K.); 4 Leibniz Institute for Food Systems Biology at the Technical University of Munich, 85345 Freising, Germany; 5 Chair of Nutritional Systems Biology, School of Life Sciences, Technical University of Munich, Lise-Meitner-Str. 34, 85345 Freising, Germany

**Keywords:** energy intake, sweet taste, peripheral serotonin, sucrose, glucose, lactisole, sugar-sweetened beverages

## Abstract

Knowledge regarding the involvement of sweetness perception on energy intake is scarce. Here, the impact of glucose and sucrose sweetness, beyond their caloric load, on subsequent food intake and biomarkers of satiation was evaluated by co-administration of the sweet taste receptor inhibitor lactisole. A total of 27 healthy, male subjects received solutions of either 10% glucose *w*/*o* 60 ppm lactisole or 10% sucrose *w*/*o* 60 ppm lactisole. Subsequent food intake from a standardized breakfast was evaluated 2 h after receiving the respective test solution. Changes in postprandial plasma concentrations of cholecystokinin, ghrelin, and serotonin were determined over a period of 120 min, as was the body temperature. Administration of lactisole to the sucrose solution increased the energy intake from the subsequent standardized breakfast by 12.9 ± 5.8% (*p* = 0.04), led to a decreased Δ AUC of the body core temperature by 46 ± 20% (*p* = 0.01), and time-dependently reduced Δ serotonin plasma concentrations (−16.9 ± 6.06 ng/mL vs. −0.56 ± 3.7 ng/mL after sucrose administration, *p* = 0.03). The present study shows that lactisole increases energy intake and decreases plasma serotonin concentrations as well as body core temperature induced by sucrose, but not glucose. This finding may be associated with the different binding affinities of sucrose and glucose to the sweet taste receptor.

## 1. Introduction

Sweet taste is discussed as a predicting factor for subsequent food consumption [1,2,3]. In particular, the sweetness of sucrose has been hypothesized to play a role in the induction of satiety [4,5], although the molecular basis for modulating the underlying regulatory processes is unknown.

Sweetness perception is mediated by the canonical sweet taste receptor, a heterodimeric G-protein coupled receptor consisting of the two subunits T1R2 and T1R3, of which T1R3 is selectively targeted by antagonist lactisole [6]. With a stimulation of the sweet taste receptor, the G-protein subunit α-gustducin leads to an activation of gustatory nerve fibers through a signal transduction pathway, transmitting the information to the brain via the central nervous system [7]. T1R2/T1R3 chemoreceptors are not only present in the oral cavity but also in extraoral tissues, such as the gastrointestinal tract [8,9], and are thought to be involved in physiological responses to nutrients, like sugar sensing, glucose homeostasis, but also the secretion of satiety hormones, thereby contributing to maintain energy balance [10,11]. The control of food intake is pivotal to energy balance and regulated by the sensation of satiation and satiety. Whereas satiety is defined as the feeling of fullness that persists after finishing a meal, satiation leads to the termination of eating [12].

In general, the circuit of hunger, food consumption, satiation and satiety is a complex interplay between central signals from the brain and peripheral processes involved in energy homeostasis [13]. Peptide hormones derived from gastrointestinal tissues, like glucagon-like peptide 1 (GLP-1), cholecystokinin (CCK), and ghrelin, unfold their anorexigenic or orexigenic properties mainly via activation of abdominal afferent nerves like the vagus nerve [13,14]. In addition, there is increasing evidence for a regulating role of peripheral serotonin not only in gastric motility but also in satiety [15]. Moreover, diet-induced thermogenesis has been shown to correlate with satiety in lean women over 24 h after receiving a high carbohydrate/protein or high fat diet [16].

The sweet taste receptors interact with the above mentioned satiety signaling pathways in several ways: GLP-1 secretion, stimulated by glucose or the non-caloric sweetener sucralose, was blocked in human enteroendocrine NCI-H716 cells in the presence of the T1R3 antagonist lactisole or siRNA against α-gustducin, which was confirmed in mice as well [9]. Moreover, intragastric as well as intraduodenal co-administration of lactisole and glucose to healthy subjects reduced the release of the satiety hormones GLP-1 and PYY compared to administration of glucose solely, indicating T1R2/T1R3 is involved in glucose-dependent secretion of satiation peptides [17]. Another in vitro study showed that caloric as well as non-caloric sweeteners induce serotonin secretion in human gastric tumor cells (HGT-1) also via targeting T1R3 [18].

Involvement of T1R2/T1R3 in diet-induced thermogenesis has not been reported yet. However, consumption of sucrose-sweetened soft cheese induced a higher thermogenic signal than soft cheese with calorie-adjusted maltodextrin or maltodextrin in combination with aspartame [19]. This supports substrate-specific effects for sucrose in diet-induced thermogenesis, which might be related to the combination of sweetness and the structure of sucrose. Therefore, the sensory profile of foods can be considered as a gatekeeper, modulating the desire and intake of food based on the sweet taste perception [20].

However, knowledge regarding the impact of the sweet intensity of, e.g., carbohydrates, on energy intake remains scarce. Glucose and sucrose are both associated with sweet taste, with sucrose showing a higher affinity for the T1R3 sweet taste receptor subunit, whereas glucose, tasting half as sweet [21], has a higher affinity for the T1R2 subunit [22]. In this study, we investigated the impact of lactisole, a T1R3 antagonist, on short-term energy intake and release of satiety hormones induced by glucose or sucrose administered to healthy subjects in concentrations corresponding to a regular soft drink.

## 2. Materials and Methods

### 2.1. Study Population

The interventional part of the human study was conducted between June 2019 and October 2019 at the University of Vienna. Volunteers were recruited using web advertising and handbills on university billboards, beginning in June 2019. Eligible for study procedure were healthy male subjects between 18–45 years, with a body mass index between 18.5–29.9 kg × m^−2^, non-smoking, with no drug abuse, and chronic diseases, and a self-reported normal sense of olfaction and sweet taste, which was confirmed by a threshold test according to DIN EN ISO146 3972:2013-12. Women were excluded of the study due to hormonal variations based on menstrual cycle [23].

To assess the state of health of the subjects, a medical screening was conducted prior to the first intervention, at which participants gave their written consent after detailed information of the intervention and data privacy guidelines. To ensure physiological response to glucose consumption, a standard oral glucose tolerance test (oGTT) with 75 g of glucose was performed following a 12 h fasting period. Urine and blood glucose concentrations were quantitated 60- and 120-min post oGTT. Additionally, a finger-prick blood test was conducted at time points t_0min_, t_15min_, t_30min_, t_60min_, t_90min_, t_120min_. Hepatic aspartate aminotransferase, alanine aminotransferase alkaline phosphatase, and γ-glutamyl transpeptidase enzyme activities and blood lipids (triglycerides, total, LDL, and HDL cholesterol) as well as thyroid hormones were analyzed by Ihr Labor 1220 (Medical diagnostics laboratory including microbiology, Dr. Gabriele Greiner, Vienna, Austria). Body weight was determined by means of a digital scale to the nearest of ±100 g (Soehnle, Nassau Germany), and body height was assed using a stadiometer (Seca213, Hamburg, Germany).

Of 39 subjects screened, 29 complied with the inclusion criteria, and 28 subjects finished all four interventions, as one subject dropped out for personal reasons. The final calculation of the results was based on 27 subjects since one subject had to be excluded due to obvious violation of the study protocol. The study population characteristics are given in Table 1.

Before starting the intervention, the study protocol was reviewed and approved by the local Ethics Board of the University of Vienna (reference number 0043).

### 2.2. Study Design and Intervention

**Figure 1:**
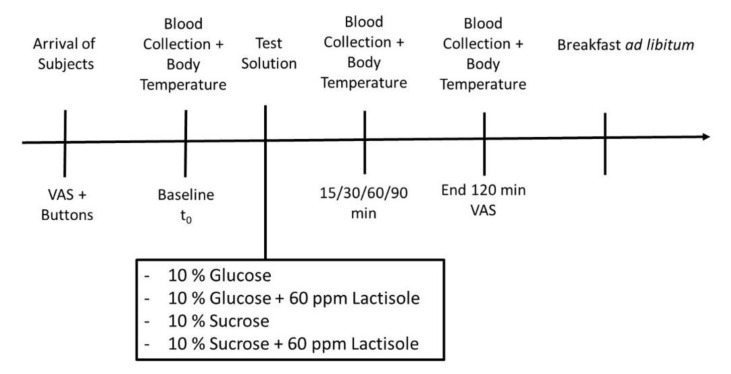
Study design of the intervention cross-over trial. A total of 27 health young men completed the four intervention days in a randomized order, either receiving a 10% glucose solution *w*/*o* 60 ppm lactisole, or a 10% sucrose solution *w*/*o* 60 ppm lactisole.

The intervention study was designed as a single blinded, randomized, controlled, monocentric, cross-over study with the following four treatments: 300 mL water + 10% (*w*/*v*) glucose (Glu), 300 mL water + 10% (*w*/*v*) glucose + 60 ppm lactisole (Glu + Lac), 300 mL water + 10% (*w*/*v*) sucrose (Suc) or 300 mL water + 10% (*w*/*v*) sucrose + 60 ppm lactisole (Suc + Lac) solutions (Figure 1). The test subjects received the treatments in a randomized order on four consecutive study days, with at least five days between each study day. All the test drinks were isocaloric (50 kJ/120 kcal). To reduce the sweetness level of the administered solutions, lactisole was added since this compound has been demonstrated to reduce the sweet taste perception by targeting the sweet taste receptor subunit T1R3 [6,24]. The concentration of 60 ppm lactisole was selected to adjust the sweetness of the 10% sucrose solution to the sweetness of the 10% glucose solution. On each study day, subjects arrived at the study site in the morning after 12 h of fasting. On each intervention day, the subjects’ feeling of hunger, energy intake after receiving the test solutions, and skin and body core temperature as well blood concentrations of ghrelin, CCK, and serotonin over a period of 120 min were assessed.

#### 2.2.1. Subjects’ Rating of Hunger

Test subjects completed a visual analogue scale (VAS), thereby reporting their subjective feeling of hunger before and 120 min after receiving the respective test solution. The VAS was designed as a 10 cm non-structured, ascending scale, starting at 0 for “not hungry at all” to 10 cm “extremely hungry”.

#### 2.2.2. Total Energy Intake

Total energy intake from a standardized, *ad libitum* breakfast of about 11.3 MJ, consisting of 46% carbohydrates, 41% fat, and 13% proteins was determined 120 min after complete swallowing of the individual test solution. The breakfast consisted of four rolls, three slices of bread, 80 g of butter, 60 g of honey, 100 g of strawberry jam, 4 slices of cheese (~125 g), 4 slices of ham (~95 g), 180 g of wild berry yoghurt, 200 mL coffee or tea, 20 g of sugar, 40 g of coffee creamer, and 200 mL of water. For subjects following a vegetarian or vegan (*n* = 2) diet, isocaloric alternatives, e.g., plant-based cheese and sausages, were provided.

Quantitative energy consumption was assessed by back weighing the non-consumed food and subtraction of the packaging weight. Calculation of food intake was performed using nut.s v1. 32.50 software.

### 2.3. Blood Sample Collection

For evaluation of plasma concentrations of postprandial hormones, venous blood was collected at six different time points by using a venous catheter as described previously [25,26,27]. The first blood drawing (t_0_) as baseline level was done in a fasted state, followed by blood drawings 15-, 30-, 60-, 90- and 120-min post intervention (Figure 1). For the quantitative analysis of ghrelin, cholecystokinin (CCK), and serotonin plasma concentrations, whole blood was collected in EDTA-coated tubes (Monovettes, Sarstedt, Nümbrecht, Germany) and centrifuged for 15 min at 1800× *g* at 4 °C. To maintain ghrelin stability, a serine protease inhibitor AEBSF (4-[2 aminoethyl benzene] sulfonyl fluoride; Merck Milipore, Darmstadt, Germany) was added before centrifugation, and plasma samples were additionally stabilized with 5% (*v*/*v*) 0.1 N hydrochloric acid. Serotonin samples were additionally centrifuged for 1 min at 7000× *g* at 4 °C to remove blood platelets. After centrifugation, all plasma samples were collected and stored at −80 °C until further analysis.

### 2.4. Determination of Postprandial Ghrelin, CCK, and Serotonin Plasma Concentrations

Measurements of ghrelin plasma (LOD 30 pg × mL^−1^) concentrations were performed by means of a sandwich enzyme linked immune assay purchased from Merck Millipore, Germany. Plasma CCK (Merck Millipore, Darmstadt, Germany LOD 0.2 pg mL^−1^) and serotonin (DLD diagnostics, Hamburg, Germany) concentrations were assessed by means of competitive ELISA kits. Serotonin samples below the detection limit were re-analyzed using the High-Sensitive Serotonin ELISA (LOD 0.39 ng/sample) purchased from DLD Diagnostics, Germany. All assays were performed according to manufacturer’s protocol.

### 2.5. Body Core and Skin Temperature

Changes in body core temperature following the carbohydrate intake as a marker for diet-induced thermogenesis were analyzed using an infrared ear thermometer IRT6520 (Braun) parallel to the blood drawing time points t_0min_, t_15min_, t_30min_, t_60min_, t_90min_, t_120min_ in triplicates. Body skin temperature was assessed with iButtons (Maxime Integrated, US), which were placed on the subject’s neck, the right scapula, the left hand, and the right shinbone according to the 4 ISO 9886-2004 (4-ISO) to evaluate a mean skin temperature [28]. iButtons function as an electronic communication interface and traced body skin temperature every 60 s, starting 10 min before blood sampling until the breakfast was finished.

### 2.6. Statistical Analysis

All statistical analyses and figure illustrations were performed using GraphPad Prism (version 8). A test for normal distribution of the data sets was conducted via Shapiro–Wilk test. Outliers were identified using the ROUT method with Q = 1%. Data are presented as Δ values (tx-t0) ± standard error of the mean, if not indicated otherwise.

VAS results indicating the subjects’ feelings of hunger at two time points are displayed as means ± SEM. Energy intake as well as temperature measurements were normalized to the respective control treatment without lactisole and are displayed as % of control. Time-dependent changes of postprandial hormones CCK, ghrelin, and serotonin were determined after normalization of the values to baseline levels (tx-t0) using a mixed-effect analysis with Sidak’s multiple comparison test. Skin temperature results from iButtons and body core temperature measurement were calculated as incremental area under the curve. Those data are presented as Δ values (tx-t0) ± SEM. According to the hypothesis, the impact of lactisole on sucrose and glucose-induced energy intake and associated markers was assessed by means of paired or unpaired (when outliers were identified), two-tailed Student’s t-test. Statistical significance was assumed at *p* < 0.05.

## 3. Results

### 3.1. Assessment of Subjects’ Feeling of Hunger by Means of Visual Analogue Scale

**Figure 2:**
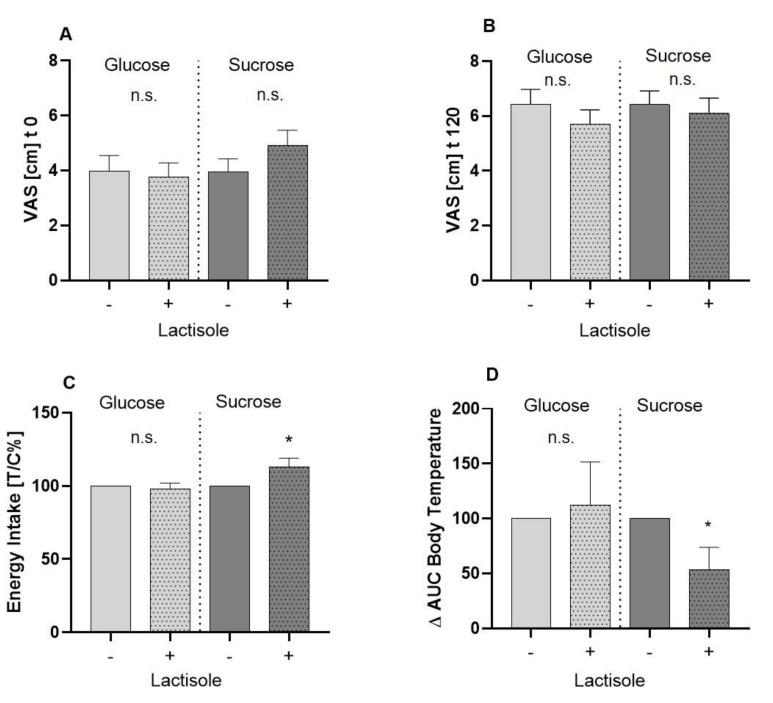
Results of food intake parameters. (**A**) Mean values ± SEM of self-reported hunger perceptions assessed by visual analog scale before and 120 min; VAS: visual analogue scale. (**B**) after administration of interventional treatments, whereby + indicates an additionally 60 ppm lactisole administration. Statistical difference (*p* < 0.05) was tested by a paired Student’s t-test (two tailed). (**C**) Results of the total energy intake of a standardized breakfast, 120 min after receiving the test solutions. All results are presented as means ± SEM treated over control. Statistical analysis (*p* < 0.05) was conducted using a paired, two tailed Student’s t-test and significant differences are marked with * *p* < 0.05. (**D**) Results of body temperature measurements represented as Δ AUC. Temperature was measured prior to the administration of test solution and 15, 30, 60, 90, and 120 min afterwards. Statistical analysis (*p* < 0.05) was conducted using a paired, two tailed Student’s t-test, and significant differences are marked with * *p* < 0.05.

The reported feelings of hunger assessed by means of a 0–10 cm VAS before (A) and 120 min after (B) administration of test solutions are presented in Figure 2. Hunger ratings differed neither before administration of the 10% glucose (4.0 ± 0.5 cm) and 10% glucose + lactisole solution (3.8 ± 0.5 cm; *p* > 0.05) (Figure 2A), nor 120 min after (Glu 4.0 ± 0.5 cm; Glu + Lac 5.7 ± 0.5 cm) administration of the study solutions. Similarly, there was no difference in hunger rating before (Figure 2A) or after the interventions with 10% sucrose and 10% sucrose + 60 ppm lactisole (Figure 2B). Mean hunger rating before the administration of sucrose + lactisole was 4.9 ± 0.5 cm, and for sucrose 4.0 ± 0.5 cm, reaching 6.4 ± 0.5 cm for sucrose and 6.1 ± 0.5 cm for sucrose + lactisole after 120 min.

### 3.2. Total Energy Intake after an Ad Libitum Breakfast

The test subjects received a standardized breakfast 2 h post-administration of the test solution and were asked to eat to a pleasant saturation level. Figure 2C shows the impact of lactisole on energy intake following oral administration of 10% glucose or 10% sucrose. Lactisole administration led to no significant difference in subsequent energy intake following glucose treatment (Glu 1226 ± 68 kcal vs. Glu + Lac 1193 ± 75 kcal, *p* > 0.05). However, lactisole administered in combination with 10% sucrose led to an increase of energy intake by 12.9 ± 5.8% (*p* = 0.04) compared to the 10% sucrose (control = 100%). The total amount of consumed kcal was 1147 ± 64 for the intervention with the sucrose solution and 1236 ± 63 kcal for the sucrose + lactisole intervention.

### 3.3. Body Core and Body Skin Temperature

Evaluation of body skin temperature by means of iButton measurement showed no significant differences between the intervention groups (data not shown). However, the incremental AUC of the body core temperature was decreased by 46 ± 20% (*p* = 0.01) after the administration of lactisole in combination with sucrose (Figure 2D), but not glucose.

### 3.4. Plasma Concentrations of Ghrelin, CCK, and Serotonin

**Figure 3:**
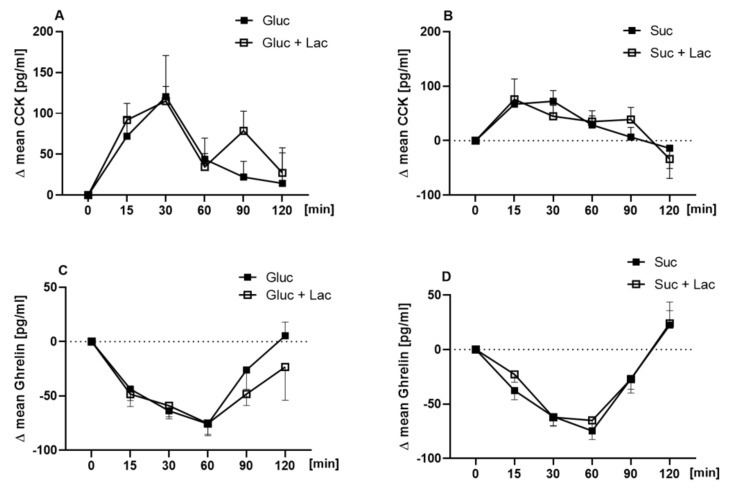
Time-dependent plasma concentrations of cholecystokinin (CCK) and ghrelin concentrations following the oral administration of 10% glucose (**A**,**C**, Glu) vs. 10% glucose + lactisole (Glu + Lac) and 10% sucrose (**B**,**D**, Suc) vs. 10% sucrose + 60 ppm lactisole (Suc + Lac). Results are presented as means ± SEM. For statistical analysis, a mixed-effect analysis with Sidak’s multiple comparison test for time and treatment was conducted.

The postprandial plasma concentrations of the hunger hormone ghrelin and the satiety hormone CCK were assessed before (t_0_) and at t_15min_, t_30min_, t_60min_, t_90min_, and t_120min_ after administration of the test solutions. Application of lactisole in combination with neither 10% glucose nor sucrose resulted in differences in postprandial CCK (Figure 3A,B) and ghrelin release (*p* > 0.05, Figure 3C,D).

**Figure 4:**
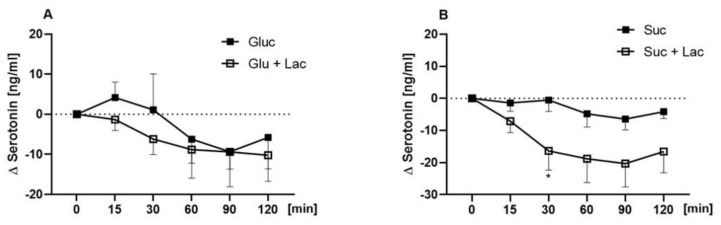
Time dependent differences in postprandial serotonin levels are presented as Δ means ± SEM for 10% glucose (Glu, **A**) vs. 10% glucose (Glu + Lac) + 60 ppm lactisole 10% sucrose (Suc, **B**) vs. 10% sucrose + 60 ppm lactisole (Suc + Lac). Statistically significant differences (*p* < 0.05) were analyzed using a mixed-effect analysis with Sidak’s multiple comparison test. Significant time dependent difference between 10% sucrose and 10% sucrose + lactisole is marked as * for *p* < 0.05.

As presented in Figure 4A, plasma serotonin concentrations did not differ between 10% glucose and 10% glucose + 60 ppm lactisole at any time point. However, serotonin concentrations showed a time-dependent difference between the sucrose + lactisole and the sucrose treatment: serotonin concentrations were higher 30 min after receiving the sucrose solution (−0.56 ± 3.7 ng/mL) than after receiving sucrose + 60 ppm lactisole (−16.9 ± 6.06 ng/mL, *p* = 0.03, Figure 4B).

## 4. Discussion

The aim of this crossover human intervention study was to investigate if the sweetness of glucose and sucrose has an impact on subsequent food intake and outcome measures of satiation and satiety since individuals’ sweet taste threshold is discussed as a modulator for dietary intake [29]. However, metabolic processes modulated by non-caloric sweeteners based on their induced sweet sensation are still under discussion [30].

We demonstrated that the concomitant application of moderate amounts of the sweet taste receptor antagonist lactisole and sucrose, but not glucose, increases subsequent energy intake of healthy young males and decreases peripheral serotonin as well as body temperature when sucrose and glucose were administered as 10% (*w*/*v*) water solutions, corresponding to sugar concentrations found in regular soft drinks or fruit juices. By co-administration of the T1R3-antagonist lactisole [6], the sweet intensity of the carbohydrate test solutions was reduced while maintaining the caloric load.

Subjective feelings of hunger by means of a VAS before and 120 min after any intervention did not change. This result is in accordance with study results from Anderson et al. [31] who showed that appetite ratings after equicaloric glucose or sucrose treatments had no different scores, even though the subsequent energy intake was divergent in effect size. Moreover, in the present study, the reported feelings of hunger did not reflect the actual total energy intake of the test subjects, as assessed by means of an ad libitum breakfast 120 min after receiving the test solution: Test subjects consumed 12.9 ± 5.8% (*p* = 0.04) more energy from the served standardized *ad libitum* breakfast when lactisole was added to the sucrose drink. In contrast, energy intake after receiving the glucose + lactisole intervention compared to glucose treatment solely showed no significant difference. This result suggests a specific effect of lactisole on sucrose-mediated molecular mechanisms, which argues against a general effect of overall sweetness, since an effect on glucose-induced energy intake could be expected then as well. Results from Anderson et al. [31] support this hypothesis, since administration of a sucrose drink reduced subsequent energy intake in comparison with application of an equi-sweet drink containing the non-caloric sweetener sucralose. Notably, distinct T1R2/T1R3 binding affinities have been demonstrated for glucose and sucrose: Whereas binding studies revealed lower glucose KD values for the T1R2 subunit, lower KD values for the T1R3 subunit were reported for sucrose [22].

To shed light on the underlying mechanisms regulating energy intake, markers associated with satiety were investigated in a period of 120 min post-administration of the carbohydrates with or without the addition of lactisole. First, body core and skin temperature as markers for diet-induced thermogenesis were investigated. Diet-induced thermogenesis is defined as an increased rate of energy expenditure following food intake and is associated with sensory profiles as well as protein [16] and carbohydrate intake [16,32]. Moreover, increased satiety has been related to diet-induced thermogenesis in lean women [16]. In the present study, we evaluated the effect of glucose and sucrose with or without co-administration of the T1R2/T1R3 antagonist lactisole on their thermogenic properties in healthy men. Two different approaches to study thermogenesis were applied: Temperature measurements in the ear to assess body core temperature and on the subject’s skin at four specified areas. While the skin temperature measurements showed no difference, the incremental AUC received from the body core temperature after consumption of the sucrose + lactisole solution was lower after the administration of 10% sucrose alone. However, results of the glucose intervention with or without lactisole revealed no differences in body temperature measurements. These findings also indicate a sucrose-specific effect of lactisole on body core temperature and may have contributed to the increased energy intake after consumption of sucrose with lactisole in comparison to sucrose alone. A sucrose-specific effect is also supported by results from Prat-Larquemin and colleagues [32] who demonstrated a higher diet-induced thermogenesis ensued by intervention with sucrose in comparison to non-sweet tasting maltodextrin and concomitant administration of maltodextrin and the low-energy sweetener aspartame in healthy male subjects.

For further elucidation, additional biomarkers of satiation, namely, plasma concentrations of CCK, ghrelin, and serotonin, were analyzed over time. The gut–brain axis is crucial for maintaining energy homeostasis. Peptides involved in the regulation of food intake in the brain are also found in the enteric nervous system and enteroendocrine cells of the mucosa of the gastrointestinal tract [13,14]. However, neither ghrelin nor CCK plasma concentrations were influenced by lactisole administration in comparison to administration of the carbohydrate solely. Moreover, Gerspach and colleagues [17] found no effect of 450 ppm lactisole on a 75 g glucose-stimulated release of CCK, whereas GLP 1 and PYY plasma concentrations were reduced.

In the present study, plasma concentrations of serotonin induced by sucrose, but not glucose, were lower when 60 ppm lactisole were applied concomitantly. The involvement of the neurotransmitter serotonin in regulating hunger and satiety in a central brain context is well established [33]. Whereas the body’s main serotonin pool is located in the periphery, e.g., in enterochromaffin cells of the gut [34], the crucial role of serotonin in a postprandial context is increasingly acknowledged: Even though serotonin displays an inability to cross the blood–brain barrier, in vivo experiments point to an anorexigenic potential of peripheral serotonin [27,35,36]. In our study, reduced serotonin plasma concentrations underline the higher intake of total energy from the standardized breakfast after receiving the sucrose + lactisole solution compared to sucrose application. Such an association between serotonin plasma levels and energy intake in men has been shown before [37]. In addition, the results of this study find support in recent in vitro findings that showed an increased serotonin release in human gastric parietal cell line after exposure to caloric or non-caloric sweeteners. Co-incubation experiments with 50 µM lactisole and a *TAS1R3* knock-down demonstrated an involvement of the T1R3 subunit in serotonin release induced by sweeteners [18]. Further, serotonin reuptake transporters (SERTs), which regulate extracellular serotonin levels [33], may contribute to taste perception processes as demonstrated by a sensory study [38]. More specifically, Heath and colleagues demonstrated SERT antagonists to modulate thresholds for sweet taste: Administration of serotonin reuptake inhibitors decreased sweet taste thresholds of a 5% sucrose solution by 27% [38]. Based on these results, lactisole might be hypothesized to modulate serotonin reuptake, possibly via antagonizing T1R3, resulting in a decreased serotonin plasma concentration.

Overall, our results suggest a pronounced role of the T1R3 subunit in processes regulating energy intake. Our main finding, showing that administration of lactisole together with sucrose, but not glucose, increased total energy intake, decreased plasma serotonin concentrations and body core temperature, might be explained by different taste receptor binding affinities. Glucose and sucrose are known to bind to the venus flytrap domain of both sweet taste receptor subunits T1R2 and T1R3 [11,22]. Sucrose was shown to bind to the T1R3 subunit with a higher affinity than glucose, which binds reversely with higher affinity to the venus fly trap domain of the T1R2 subunit [11]. Since lactisole is known to specifically target the T1R3 subunit as well, the stronger effect of lactisole on sucrose-induced energy intake may thus be based on higher T1R3 binding affinities of sucrose and lactisole. Moreover, an interaction of sweet taste receptors and glucose-sensitive carbohydrate sensors may help to explain the differences between lactisole-sensitive signaling of glucose and sucrose. Activation of the sweet taste receptor by sugars or sweeteners has been shown to promote glucose uptake via increased expression of sodium-dependent glucose transporter isoform 1 (SGLT-1) [39,40], which has been associated with increased incretin hormone release [9], linking SGLT-1 to satiation signals. In addition, SGLT-1 and other glucose transporters, as well as components of the ATP-gated K+ channels are also expressed in taste cells and are thought to contribute to the sweet taste of sugars in the absence of the sweet taste receptor [41]. Sucrose is cleaved into its two components, fructose and glucose, by salivary or intestinal glucosidases; however, fructose, in contrast to glucose, is not transported by SGLT-1 [42]. Thus, when applied in equal amounts, sucrose is likely to activate SGLT-1-mediated satiety responses to a smaller extent as the same amount of glucose when the sweet taste receptor is blocked by lactisole. The findings of the present study might thus reflect a cross-talk between the sweet taste receptors, SGLT-1 and satiety signals.

This study has potential limitations. First, the unknown effects of lactisole administration on long-term energy intake remain unclear. In addition, to the best of our knowledge, this is the first study to investigate the impact of the T1R3 antagonist lactisole on energy intake, and the underlying mechanisms can only be assumed based on previous in vitro studies on individual satiety markers. In addition, it cannot be excluded that lactisole administered in concentrations higher than 60 ppm, completely blocking sweet taste perception [43], has different effects on molecular pathways including glucose-induced responses. Moreover, no female subjects were included in the study, which needs to be addressed in future studies.

## 5. Conclusions

The present study shows that lactisole increases energy intake and decreases plasma serotonin concentrations as well as body core temperature induced by sucrose, but not glucose, when administered to healthy male subjects in dietary relevant concentrations. A greater understanding of the biomolecular mechanism followed by sweet ingestion and sweet taste receptor involvement in the oral and gastrointestinal tract is needed.

## Figures and Tables

**Table 1:** Study subjects’ characterization

*n*	27
Gender	Male
Age [years]	28 ± 5
Body Weight [kg]	77.4 ± 12
Height [m]	1.81 ± 0.1
BMI [kg/m^2^]	23.8 ± 2.7

Data are presented as means ± SEM. BMI: body mass index.
